# AM-MTEEG: multi-task EEG classification based on impulsive associative memory

**DOI:** 10.3389/fnins.2025.1557287

**Published:** 2025-03-06

**Authors:** Junyan Li, Bin Hu, Zhi-Hong Guan

**Affiliations:** ^1^School of Future Technology, South China University of Technology, Guangzhou, China; ^2^Guangdong Artificial Intelligence and Digital Economy Laboratory, Guangzhou, China; ^3^School of Artificial Intelligence and Automation, Huazhong University of Science and Technology, Wuhan, China

**Keywords:** electroencephalogram (EEG), brain-computer interface, bidirectional associative memory, impulsive neural network, multi-task learning

## Abstract

Electroencephalogram-based brain-computer interfaces (BCIs) hold promise for healthcare applications but are hindered by cross-subject variability and limited data. This article proposes a multi-task (MT) classification model, AM-MTEEG, which integrates deep learning-based convolutional and impulsive networks with bidirectional associative memory (AM) for cross-subject EEG classification. AM-MTEEG deals with the EEG classification of each subject as an independent task and utilizes common features across subjects. The model is built with a convolutional encoder-decoder and a population of impulsive neurons to extract shared features across subjects, as well as a Hebbian-learned bidirectional associative memory matrix to classify EEG within one subject. Experimental results on two BCI competition datasets demonstrate that AM-MTEEG improves average accuracy over state-of-the-art methods and reduces performance variance across subjects. Visualization of neuronal impulses in the bidirectional associative memory network reveal a precise mapping between hidden-layer neuron activities and specific movements. Given four motor imagery categories, the reconstructed waveforms resemble the real event-related potentials, highlighting the biological interpretability of the model beyond classification.

## 1 Introduction

The brain-computer interface (BCI) can be defined as a system that translates a user's brain activity patterns into messages or commands for interactive applications (Lotte et al., 2018). Efficient BCI systems can promote interactions between the brain and physical devices and have broad applications in medical rehabilitation and neuroscience research (Lebedev and Nicolelis, 2017). Most of the current BCI data comes from neural electrical signals recorded by electroencephalogram (EEG), which enables researchers to measure and decode human brain activity. A classic BCI paradigm for motor imagery (MI) is consisted of five parts: EEG acquisition, EEG preprocessing, feature extraction, classification, and task execution (Lotte et al., 2015). One crucial step is to extract features of EEG signals and classify them into the motion categories. To date, the EEG classification tasks are still constrained by the following limitations.

- Since the EEG data has large variability and the representation of neural activity changes over time (Degenhart et al., 2020), EEG classification models trained on given sample datasets are difficult to generalize to other samples.
- With the development of biosignal sensors for use in home environments (Zhang et al., 2023), processing models for biosignals require increasingly robust performance. However, most EEG classification models have low cross-subject classification accuracy (Lawhern et al., 2018), where models are only trained and tested on a single subject's data (Hu et al., 2019b), resulting in low generalization in cross-subject tasks.
- Because BCI experiments are time-consuming (Shen et al., 2022), and are limited by the energy and time of the subjects, the amount of EEG data collected by a single subject is relatively small, detrimental to the training of computational models.

Data-driven deep learning has achieved remarkable achievements in image classification, speech recognition, and natural language processing, among others (Ma et al., 2020). Due to the above-mentioned limitations, however, current deep learning models cannot be directly applied to deal with the EEG classification tasks, particularly for the cross-subject issue. In view of the ethical and safety considerations, the healthcare field has raised high requirements on the interpretability of deep learning models (Adadi and Berrada, 2018). Most of the existing models have poor interpretability given the end-to-end scenario, limiting their application in BCI. It is thus in demand to build new machine learning models to fix the cross-subject variability of EEG by data sharing, and enhance the neuroscience interpretability for wide application to BCI systems.

**Figure 1:**
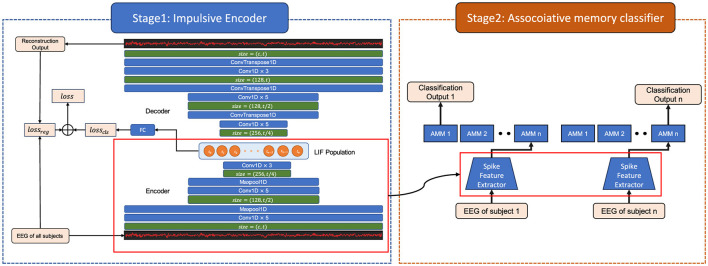
The architecture of the associative memory multi-task EEG (AM-MTEEG) model. There are two stages in the model. Stage 1 includes a convolutional encoder, an impulsive neural population, a decoder of transposed convolutions. Stage 2 is a bidirectional associative memory (BAM) classifier. Both the convolution and transposed convolution layers use a convolution kernel of length 5 and are activated through the ReLU function. The impulsive neural population is built with 200 leaky integrate-and-fire (LIF) neurons. The encoder, population and decoder are trained by backpropagation (BP) with joint loss, while the BAM module is optimized by Hebbian learning.

This article resorts to the multi-task learning (MTL) to cope with the large variability in the EEG data. The EEG classification of each subject is defined as a task, common features are extracted among samples from various subjects to guarantee cross-subject training, and these features are mapped into categories of each subject. Differing from the closely-related methods (Zheng et al., 2019; Wang et al., 2024; Lawhern et al., 2018), we incorporate associative memory to multi-task learning and propose the AM-MTEEG model by mixing Hebbian learning with deep learning (Figure 1). The AM-MTEEG combines a deep convolutional model with an impulsive associative memory network, as inspired by the memory principle of human brains (Seitz, 2010), which can learn accurate mappings from very few demonstrations. AM-MTEEG includes a deep learning encoder-decoder counterpart trained across various samples, replacing the encoding mechanism of the brain. The model further incorporates a layer of impulsive neurons to encode the necessary neural signals for associative memory formation. For each subject, a bidirectional associative memory network is built to map impulsive signals to the motion categories. The original EEG signals can be reconstructed by decoding the impulsive signals associated with specific category labels, thereby enhancing the interpretability beyond the classification process.

The main contributions of this article are summarized as follows.

- To deal with the cross-subject variability, a multi-task EEG classification model is proposed, termed as AM-MTEEG, which integrates a deep learning-based convolutional encoder with a Hebbian-type bidirectional associative memory (AM) network. The convolutional encoder captures shared features across different samples, while the AM network alleviates the data variability for classification by directly mapping latent features to motion categories.
- AM-MTEEG achieves an average accuracy of 86% on the BCI Competition IV IIa dataset, surpassing state-of-the-art (SOTA) methods and exhibiting minimal performance variance across different samples. The decoder can reconstruct EEG signals from neural activities in the AM network, which resembles the real event-related potentials (ERPs) of each motion, thereby demonstrating the model interpretability.
- For any motor imagery EEG, impulsive neurons in AM-MTEEG exhibit specific firing patterns characterized by high synchrony. This firing synchrony suggests that neuronal activity is governed by a low-dimensional latent manifold, a feature consistent with the neural coding mechanisms observed in hippocampal neurons (Levy et al., 2023). This alignment indicates the neuro-inspired characteristic of the model, reflecting the biological plausibility.

## 2 Related work and research motivation

In EEG-based BCI tasks, one common approach is to utilize common spatial patterns (CSP) for feature extraction (Blankertz et al., 2007), followed by classification algorithms like LDA (linear discriminant analysis) and SVM (support vector machine). Although CSP helps to extract EEG features, traditional machine learning models are not adequate to recognize complex EEG patterns. Current deep learning models have demonstrated greater flexibility in EEG classification for complex BCI tasks (Lawhern et al., 2018; Altaheri et al., 2022). For example, Lawhern et al. (2018) proposed EEGNet, which applies separable two-dimensional convolutions to EEG classification problems. Liu et al. (2023) combine the same spatiotemporal convolution with filter banks and propose FBMSNet, which mixes deep convolution to extract temporal features at multiple scales and then performs spatial filtering to mitigate volume conduction. Altaheri et al. (2022) introduced ATCNet, a convolutional neural network with temporal attention mechanisms for EEG classification. ATCNet achieved an average accuracy of 85.4% on the BCI Competition IV IIa dataset, setting a new state-of-the-art performance on this dataset.

The accuracy of BCI decoding across subjects is constrained by the variability of emotion and experience between subjects (Huang et al., 2023). Waytowich et al. (2016) presented a spectral transfer model using information geometry to sort and combine the prediction results of information geometry classifier sets to achieve unsupervised transfer learning for single test detection. Zhi et al. (2024) proposed a generalization network using domain alignment and class regularization blocks of deep correlation alignment to establish domain-independent supervised contrastive learning. Another solution comes from multi-task learning, which allows different tasks to share common features. Compared to single-task learning models, MTL leverages more data from different tasks (Zhang and Yang, 2021), enabling the learning of more generalized representations. Moreover, MTL can be used to alleviate large high subject variability and limited sample size in EEG-based BCI tasks. Zheng et al. (2019) developed an effective algorithm where each subject's sample is treated as a separate task, utilizing regularized tensors. In addition to MTL, the use of ensemble learning can also reduce the variability of EEG. For example, Qi et al. (2022) used a dynamic ensemble Bayesian filter to assemble models to cope with variability in signals.

Spiking neural networks (SNNs), inspired by neural systems in the brain, offer computational advantages such as low power consumption and high interpretability, making them widely applicable across various tasks. Diehl and Cook (2015) implemented an SNN using unsupervised STDP learning for handwritten digit classification, achieving 95% accuracy on the MNIST dataset. Xu et al. Ma et al. (2020) used a spiking recurrent neural network to extract features and presented a neuromorphic approach for classifying electromyography (EMG) signals. Xu et al. (2023) proposed a spiking convolutional neural network for electromyography pattern recognition, which can be used in prosthesis control and human-computer interaction. The effectiveness of SNNs in multi-task learning has also been demonstrated. For instance, Cachi et al. (2023) proposed TM-SNN, which uses different spiking thresholds to represent different tasks while sharing the same structure and parameters across tasks. Convectional spiking networks cannot be used for EEG-based BCI tasks, since the training process would be hampered by the limited data and large variability across subjects.

Previous studies suggest that hybrid models that integrate associative memory networks with deep learning could perform better than conventional convolutional or spiking networks across tasks. Hu et al. (2019a) proposed that spiking neural networks using Hebbian learning can provide stable and fault-tolerant associative memory. Miconi et al. (2018) combined Hebbian rule-based associative memory with traditional backpropagation neural networks, achieving efficient learning on small-sample image datasets. Wu et al. (2022) applied a similar structure to spiking neural networks, where the network weights are updated through both global learning via backpropagation and local Hebbian learning. This hybrid method performed well in fault-tolerant learning and few-shot learning. The associative memory networks (Hu et al., 2019a; Kosko, 1988) could yield a short training time by directly mapping the spike representations to motion categories, thus reducing the calibration time of BCI systems on new subjects.

Based on these observations, this article develops a hybrid model of deep convolutional network and impulsive associative memory network, for the purpose of extracting cross-subject features. The training of the AM network has a linear time complexity for reducing the training time, while deep convolutional networks have flexible feature extraction capabilities. Moreover, the AM network can enhance the interpretability of BCI performance across different subjects, while the shared features extracted through multi-task learning improve classification stability across subjects. As a by-product, the proposed hybrid model enables to share data across subjects and reduce the time to adapt to new subjects, which could be useful for dealing with limited data. When used for EEG-based BCI systems, the combination of AM and deep learning helps to cut the calibration time onto new subjects and lessen the amount of data required for a single subject within a cross-subject dataset.

## 3 The AM-MTEEG model

Figure 1 demonstrates the proposed associative memory multi-task EEG (AM-MTEEG) model, consisting of an impulsive encoder and an associative memory classifier. The impulsive encoder utilizes a one-dimensional convolutional neural network to extract signal features, which are then fed into a population of spiking neurons, encoding the input into low-dimensional spiking representations. A convolutional neural network decoder is employed to reconstruct the EEG signals. The associative memory classifier assigns an associative memory matrix (AMM) to each classification task, mapping the encoded spikes to multi-task categories. The training of the AM-MTEEG model entails the following two stages.

- **Stage 1:** The impulsive encoder and decoder modules combine self-supervised learning with label-guided training to optimize the parameters. Multi-channel EEG signals are used simultaneously as both input and target, training the spiking encoder to reconstruct the one-dimensional EEG signals.
- **Stage 2:** The spiking encoder employs the pre-trained parameters from Stage 1. Only the associative memory network is trained for different tasks. The input and category label are represented as an input-output pattern pair {**x**_*i*_, **y**_*i*_}, where ${\text{x}}_{i}\in R^{nt}$ is the low-dimensional spiking representation vector from the spiking encoder, *n* is the number of neurons, *t* is the length of time series from the convolution encoder, and **y**_*i*_ is the one-hot target vector. The associative memory network matches the input patterns to the corresponding output patterns by bidirectional hetero-associative memory.

### 3.1 Convolutional feature extractor

As shown in Figure 1, the convolutional module consists of an encoder *E* and a decoder *D* built with one-dimensional convolutions. The convolution kernel length of the convolutional layer is 5 and the ReLU (Rectified linear unit) function is used as activation. Unlike the existing EEG classification models based on 2D convolution (Lawhern et al., 2018; Altaheri et al., 2022), to achieve EEG data classification while maintaining the structure of the original EEG signal as much as possible, we only used 1D convolution for feature extraction. Therefore, the convolution kernel parameters to be trained are reduced from *c*×*n*^2^ to *c*×*n*, where *c* is the number of signal channels and *n* is the convolution kernel size, which allows us to use larger convolution kernels. In the motor imagery task, we used the CNN model architecture as shown in Table 1.

**Table 1:** The output size of each module in AM-MTEEG.

**Blocks**	**Layer**	***N*_*conv*_**	**Size**	**Stride**	**Activation**	**Output**
Encoder	Input		(*C, T*)			(*C, T*)
	Conv1D block	5	5	1	ReLU	(128, *T*)
	AvgPool1D		2	2		(128, *T*/2)
	Conv1D block	5	5	1	ReLU	(256, *T*/2)
	AvgPool1D		2	2		(256, *T*/4)
	Conv1D block	3	5	1	ReLU	(256, *T*/4)
Neural Population	FC		(256, 200)			(200, *T*/4)
	LIF neurons		200			(200, *T*/4)
	FC		(200, 256)			(256, *T*/4)
Decoder	Conv1D block	5	5	1	ReLU	(128, *T*/4)
	ConvTranspose1D		8	2		(128, *T*/2)
	Conv1D block	5	5	1	ReLU	(128, *T*/2)
	ConvTranspose1D		8	2		(128, *T*)
	Conv1D block	3	5	1	ReLU	(*C, T*)
Classifier	AMM		(200 × *T*/4, *N*_*class*_)			*N*_*class*_

The input signal **x** ∈ *R*^*ct*^ is downsampled to 1/4 of the original length by two one-dimensional maximum pooling in the encoder to obtain the hidden signal


(1) $$\begin{array}{l} \text{h}=E\left(\text{x}\right),\text{h}\in R^{nt/4}. \end{array}$$

The encoded signal is directly input into the spiking neuron as the current through the fully connected layer, recording the spike sequence ${\text{S}}_{p}\in R^{nt/4}$ emitted by the neuron. In the decoder stage, the hidden layer spike are mapped by the fully connected layer and then upsampled to the original length by two one-dimensional deconvolutions.


(2) $$\begin{array}{l} {\text{x}}^{\prime }=D\left({\text{S}}_{p}\right),{\text{x}}^{\prime }\in R^{ct}. \end{array}$$

In this process, the encoder-decoder module and the impulsive neural population obtain a low-dimensional representation of EEG activity through autoregressive learning.

### 3.2 Impulsive neural population

Let the encoder output signal be the current *I*, which is to be input into the leaky integrate-and-fire (LIF) neuronal population. The purpose is to convert the encoder output into a discrete spike train **s**. By the LIF mechanism (Ward and Rhodes, 2022), the membrane potential *u*(*t*) evolves as


(3) $$\begin{array}{l} \tau \frac{du}{dt}=-u+RI\left(t\right), \end{array}$$

where *R* is a constant resistance. When the membrane potential is greater than a given threshold *u*_*th*_, the neuron generates a spike, and then the membrane potential would be reset to 0. In computer simulation, we use the following differential form


(4) $$\begin{array}{l} u^{t}=\left(1-\tau \right)u^{t-1}-s^{t-1}u_{th}+\underset{i}{\sum }\left(w_{i}I_{i}^{t-1}\right),\\ s^{t}=step\left(u^{t}-u_{th}\right), \end{array}$$

where τ is the decay constant, *w*_*i*_ is the synaptic weight of the synapse *i*, *s*^*t*^∈{0, 1} is the spike fired at *t*, and $I_{i}^{t-1}$ represents the input current of the synapse *i* at time *t*−1. When the membrane potential is greater than *u*_*th*_, the neuron generates a spike, and the membrane potential is set to 0 at the next time *t*+1. As shown in Equation 4, This process uses the unit step function


$$step(x)=\{\begin{array}{ll} 1, & x\geq 0,\\ 0, & x<0. \end{array}$$

While training the encoder using backpropagation, calculating the gradient of the step function poses a challenge. Since the step function is discontinuous, its gradient results in an impulse response


$$\delta (x)=\{\begin{array}{ll} +\infty , & x=0,\\ 0, & x\neq 0. \end{array}$$

The gradient of membrane potential is calculated as


(5) $$\begin{array}{l} \nabla u^{t-1}=\frac{\partial u^{t}}{\partial u^{t-1}}\nabla u^{t}+\frac{\partial s^{t-1}}{\partial u^{t-1}}\nabla s^{t-1}\\ \;=\left(1-\tau \right)\nabla u^{t}+\delta \left(u^{t}-u_{th}\right)\nabla s^{t-1}\\ \;=\nabla u^{t}\left[\left(1-\tau \right)+u_{th}\delta \left(u^{t}-u_{th}\right)\right]. \end{array}$$

**Figure 2:**
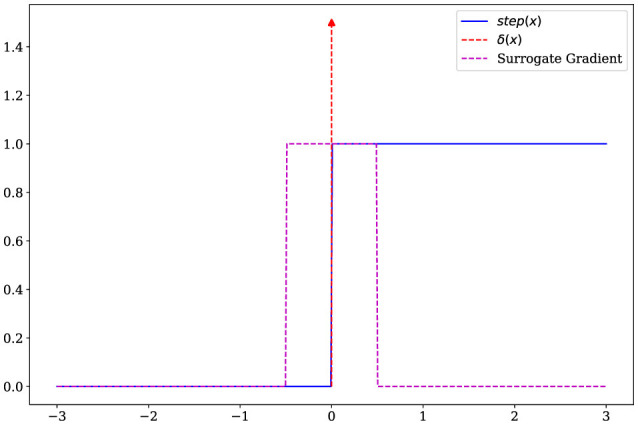
The step function, impulse function, and surrogate gradient function.

Note that, the term $\delta \left(u^{t}-u_{th}\right)$ makes it difficult to train the encoder. As shown in Figure 2, we use the surrogate gradient method (Neftci et al., 2019) to replace the impulse function with a rectangular window function


(6) $$rect(x)=\{\begin{array}{ll} 1, & |x|\leq 0.5,\\ 0, & |x|>0.5. \end{array}$$

Then, using the surrogate gradient, the membrane potential gradient can be approximated by


(7) $$\begin{array}{l} \nabla u^{t-1}=\frac{\partial u^{t}}{\partial u^{t-1}}\nabla u^{t}+{\left(\frac{\partial s^{t-1}}{\partial u^{t-1}}\right)}^{\prime }\nabla s^{t-1}\\ \;=\left(1-\tau \right)\nabla u^{t}+rect\left(u^{t}-u_{th}\right)\nabla s^{t-1}\\ \;=\nabla u^{t}\left[\left(1-\tau \right)+u_{th}rect\left(u^{t}-u_{th}\right)\right]. \end{array}$$

At each time *t*, each signal processed by the convolutional neural network is treated as an input current (Figure 1), which is passed through a fully connected layer into multiple LIF neurons in the hidden layer. The hidden layer generates spikes, and these spike sequences encode essential information from the original EEG signal. Subsequently, we employ a self-supervision approach, using a convolutional neural network decoder to reconstruct the EEG signal. To ensure the identifiability of the impulses in signal reconstruction, the hidden layer neurons are linked to an auxiliary classifier for preliminary classification. Here, we use a trainable fully connected layer, with a joint loss function of reconstruction-type *L*_*reg*_ and classification-type *L*_*cls*_. The reconstruction loss is defined by mean square error (MSE): $L_{reg}\left(\text{x},\hat{x}\right)=\frac{1}{n}\underset{i}{\sum }{\left(x_{i}-\hat{x_{i}}\right)}^{2}$, where **x** is the vector of original EEG signals of all subjects and $\hat{x}$ is the reconstructed one. The classification loss uses cross-entropy loss, i.e., $L_{cls}\left(\text{x},label\right)=-\underset{i}{\sum }label_{i}log\left(x_{i}\right)$, where *label* is the motion categories of the MI task. The joint loss function has the following format:


(8) $$\begin{array}{l} L=L_{reg}+\lambda L_{cls}, \end{array}$$

where λ > 0 is a mixing factor. In computer simulation, one has λ = 0.1, and the *L*_*cls*_ is calculated by the hidden layer spike through the auxiliary classifier. *L*_*reg*_ makes the model more expressive, while *L*_*cls*_ enhances the identifiability of the latent space. When trained with by the joint loss (Equation 8), AM-MTEEG can perform the classification and reconstruction tasks simultaneously.

### 3.3 Associative memory classifier

In order to promote efficient training and accurate classification, we build a bidirectional associative memory network as a classifier to map the impulse activities into labels of the EEG data. Let the input-output pattern (or task) pair be {**x**_*k*_, **y**_*k*_}, where ${\text{x}}_{k}\in R^{n}$ is the input column vector, ${\text{y}}_{k}\in R^{m}$ is output one-hot vector, and *k* is the task index. In the memory retrieval stage, the iterative process of the pattern pair {**x**_*k*_, **y**_*k*_} is performed as Kosko (1988)


(9) $$\begin{array}{l} {\text{y}}_{k}^{t+1}=sgn\left({\text{W}}_{k}{\text{x}}_{k}^{t}\right),\\ {\text{x}}_{k}^{t+1}=sgn\left({\text{W}}_{k}^{T}{\text{y}}_{k}^{t}\right), \end{array}$$

where ${\text{W}}_{k}=w^{ij}\in {ℜ}^{m\times n}$ is the associative memory matrix (AMM), $sgn(z)=\{\begin{array}{ll} -1 & z\leq 0\\ +1 & z>0 \end{array}$, and *t* is the time.

In the following, the index *k* is omitted for simplicity. The Equation 9 can be written as the sum of all elements:


(10) $$\begin{array}{l} y_{i}^{t+1}=\overset{n}{\underset{j=1}{\sum }}w^{ij}x_{j}^{t},\\ x_{j}^{t+1}=\overset{m}{\underset{j=1}{\sum }}w^{ji}y_{i}^{t}. \end{array}$$

When the associative memory network is stable, one has *y*^*t*+1^ = *y*^*t*^, *x*^*t*+1^ = *x*^*t*^. Then, the above process is actually optimizing the energy function of the system (Kosko, 1988)


(11) $$\begin{array}{l} E_{t}=-(y^{t}{)}^{T}Wx^{t}\\ \;=\sum_{i}\sum_{j}-y_{t}^{i}w^{ij}x_{t}^{j}. \end{array}$$

The gradient of *E*_*t*_ regarding *w*^*ij*^ is $\frac{\partial E}{\partial w^{ij}}=-y^{i}x^{j}$, and the energy function achieves its minimum by taking *w*^*ij*^ = *sgn*(*y*^*i*^*x*^*j*^).

During the training stage, the associative memory matrix **W**_*k*_ of the task *k* is


(12) $$\begin{array}{l} {\text{W}}_{k}=\underset{j}{\sum }{\text{y}}_{k}^{j}{\text{x}}_{k}^{jT}. \end{array}$$

This equation suggests that the bidirectional associative memory fits into the correlation-based Hebbian learning. When the pre and post-synaptic neurons emit spikes simultaneously, the synaptic connection would be strengthened. This process is similar to the long-term synaptic plasticity mechanism of hippocampal neurons (Kelso et al., 1986).

Next, we prove the convergence of the bidirectional associative memory network. Let $\Delta x_{t}^{j},\Delta y_{t}^{i}$ be the changes of *x*^*j*^, *y*^*i*^ at time *t*. Then, the time difference of the energy function *E*_*t*_ is


(13) $$\begin{array}{l} \Delta E_{t}=\underset{j}{\sum }\frac{\partial E_{t}}{\partial x_{t}^{j}}\Delta x_{t}^{j}+\underset{i}{\sum }\frac{\partial E_{t}}{\partial y_{t}^{i}}\Delta y_{t}^{i}. \end{array}$$

From Equation 11, it follows that


(14) $$\begin{array}{l} \frac{\partial E_{t}}{\partial x_{t}^{j}}=\underset{i}{\sum }-y_{t}^{i}w^{ij},\\ \frac{\partial E_{t}}{\partial y_{t}^{i}}=\underset{j}{\sum }-x_{t}^{j}w^{ij}. \end{array}$$

Substituting Equation 14 into Equation 13, one can get


(15) $$\begin{array}{l} \Delta E_{t}=-\underset{j}{\sum }{\left(\underset{i}{\sum }w^{ij}y_{t}^{i}\right)}^{2}-\underset{i}{\sum }{\left(\underset{j}{\sum }w^{ij}x_{t}^{j}\right)}^{2}, \end{array}$$

where


(16) $$\begin{array}{l} {\left(\underset{i}{\sum }w^{ij}y_{t}^{i}\right)}^{2}\geq 0,\\ {\left(\underset{j}{\sum }w^{ij}x_{t}^{j}\right)}^{2}\geq 0. \end{array}$$

Therefore, Δ*E* ≤ 0 holds, and the dynamic changes of the system will cause *E* to continue to decrease. Considering the use of the *sgn* function, the system will gradually converge to a stable value.

One-hot encoded **y**^*j*^ is used as the output pattern. After applying the *sgn* function, the maximum value is set to 1, while the remaining values are set to -1. This system stabilizes after a single iteration. Therefore, during the testing phase, for a given task sample **x**_*i*_, the classification result is obtained using the associative memory matrix


(17) $$\begin{array}{l} label^{i}=\underset{i}{\arg\min}\left({\text{W}}^{i}{\text{x}}^{i}\right). \end{array}$$

Considering all the pattern pairs {**x**_*k*_, **y**_*k*_} to be stored, for any input **x**_*i*_ in the prediction phase, it follows that


$${\text{y}}_{i}=\underset{k}{\sum }{\text{y}}_{k}{\text{x}}_{k}^{T}{\text{x}}_{i},$$

The above process is equivalent to taking the cosine similarity between the current input **x**_*i*_ and **x**_*k*_ in all pattern pairs as the average output **y**_*k*_ of the weighted calculation.

## 4 Experimental results

The proposed AM-MTEEG model is validated on two public datasets, fitting the classic motor imagery BCI paradigm. In the following experiments, we utilize the BCI Competition datasets and achieve an average accuracy over 94% and 86% on two of its subsets, respectively.

### 4.1 Dataset description

**BCI Competition III Iva** is a binary classification dataset, including right-hand and foot movement imagery tasks performed by 5 subjects. Each task includes 118 channels of EEG signals obtained at a sampling rate of 100 Hz within 3 s (Dornhege et al., 2004).

**BCI Competition IV IIa** is a four-category dataset, including motor imagery tasks of the left hand, right hand, feet, and tongue performed by 9 subjects. Each task includes 22 channels of EEG signals and 3 channels of EOG signals obtained at a sampling rate of 250 Hz within 3 s (Brunner et al., 2008).

### 4.2 Performance evaluation

#### 4.2.1 Comparative studies

As shown in Tables 2, 3, we compare the AM-MTEEG model with other cross-subject (CS) or inner-subject (IS) models. In contrast to SOTA methods, AM-MTEEG achieves comparable accuracy and surpasses the current SOTA in terms of average accuracy on the BCI Competition IV IIa dataset. Compared to other multi-task models, the proposed model exhibits the smallest standard deviation in accuracy across different subjects, indicating its ability to provide stable classification performance across subjects. Additionally, when extending to new tasks, AM-MTEEG only requires retraining the associative memory matrix, and the Hebbian learning used in this process is highly efficient, demonstrating its good scalability.

**Table 2:** Accuracy comparisons on BCI competition IV IIa, including cross-subject (CS) model and inner-subject (IS) model.

**Model**	**Tensor-based MTL**	**TFTL**	**EEGNet**	**ATCNet**	**Ours**
Source	Zheng et al. (2019)	Wang et al. (2024)	Lawhern et al. (2018)	Altaheri et al. (2022)	\
Type	CS	CS	IS	IS	CS
1	0.840	0.826	0.858	**0.885**	0.810
2	0.573	0.673	0.615	0.705	**0.793**
3	0.549	0.951	0.886	**0.976**	0.879
4	**0.959**	0.797	0.749	0.810	0.831
5	0.912	0.743	0.559	0.830	**0.948**
6	0.826	0.757	0.521	0.736	**0.897**
7	0.792	0.736	0.896	**0.931**	0.862
8	0.835	0.882	0.833	**0.903**	0.879
9	0.819	**0.923**	0.795	0.910	0.844
AVG	0.790	0.810	0.745	0.854	**0.860**
STD	0.131	0.066	0.139	0.086	**0.045**

Bold represents the highest accuracy or lowest standard deviation of each row.

**Table 3:** Accuracy comparisons on BCI competition III Iva, including cross-subject (CS) model and inner-subject (IS) model.

**Model**	**EEGNet**	**EDPNet**	**Tensor-based MTL**	**Ours**
Source	Lawhern et al. (2018)	Han et al. (2024)	Zheng et al. (2019)	\
Type	IS	IS	CS	CS
aa	**1.000**	**1.000**	0.911	0.958
al	0.688	0.884	**1.000**	0.975
av	0.582	0.704	0.768	**0.838**
aw	0.795	0.835	**1.000**	0.975
ay	0.516	0.679	0.929	**0.950**
AVG	0.716	0.820	0.921	**0.942**
STD	0.176	0.118	0.084	**0.052**

Bold represents the highest accuracy or lowest standard deviation of each row.

#### 4.2.2 Ablation studies

**Figure 3:**
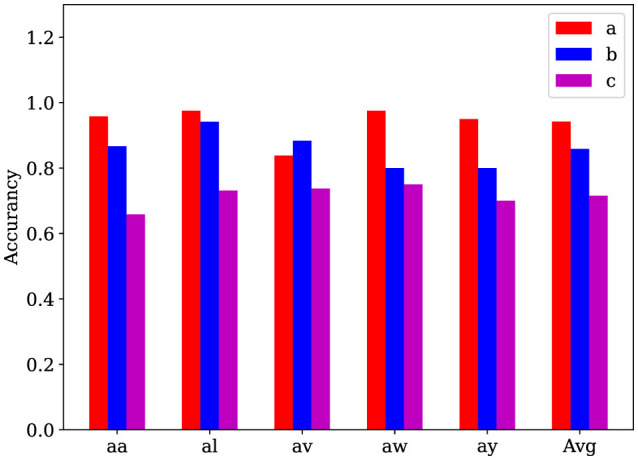
Ablation experiments on the BCI Competition III Iva binary classification dataset. (a) The full AM-MTEEG model; (b) Model with spiking neurons removed; (c) Model with a fully connected network using gradient descent instead of the associative memory classifier. These accuracy histograms show that the classification performance can be improved by incorporating the spiking neurons and the bidirectional associative memory networks in the AM-MTEEG model.

We evaluate the AM-MTEEG model on the BCI Competition III Iva dataset and compare the full model with two simplified models: (a) a model where spiking neurons were replaced with the tanh function, and (b) a model where the associative memory matrix was replaced by a fully connected layer trained via gradient descent. The results, as shown in Figure 3, demonstrate that the full model outperforms the simplified models in terms of accuracy on most samples. These findings suggest that both the spiking computation and the bidirectional associative memory classifier used in AM-MTEEG contribute to the improved performance.

### 4.3 Model interpretability

Due to the reversibility of bidirectional associative memory, the classification label is fed into the associative memory matrix to obtain the characteristic impulse sequence corresponding to any motion category. Define


(18) $$\begin{array}{l} x_{i}^{label}=sgn\left({\text{W}}_{i}^{T}{\text{y}}^{label}\right). \end{array}$$

**Figure 4:**
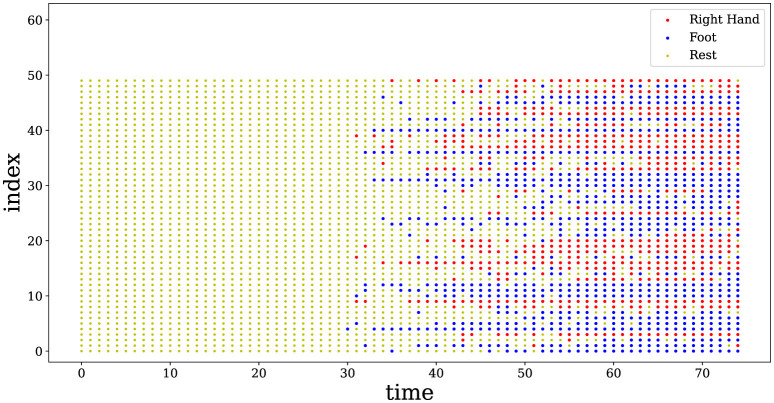
Spike raster plot of neuronal activities corresponding to the two categories of motions. 50 neurons are randomly selected from the AM network. The spikes of neurons exhibit a high degree of synchrony over time, while the synchronous behavior represents a fixed category. Red represents the spike emission with a right hand movement, and blue shows the spike emission related to a foot movement. Yellow means no spike, i.e., neurons stay in the resting state.

Figure 4 shows the impulse sequences obtained through the associative memory network, on the BCI Competition III Iva dataset. Here, we visualize the spiking neuronal activities from subject aa performing a motor imagery task with the right hand and foot, which reflects the neural coding behind the movements (Shen et al., 2023). It can be observed that the spikes exhibit a high degree of synchrony over time, and this synchronous behavior of neurons corresponds to a fixed category. This finding suggests that the impulsive neuronal population in the hidden layer fires within a similar pattern to that measured in the hippocampus of human brains (Levy et al., 2023).

**Figure 5:**
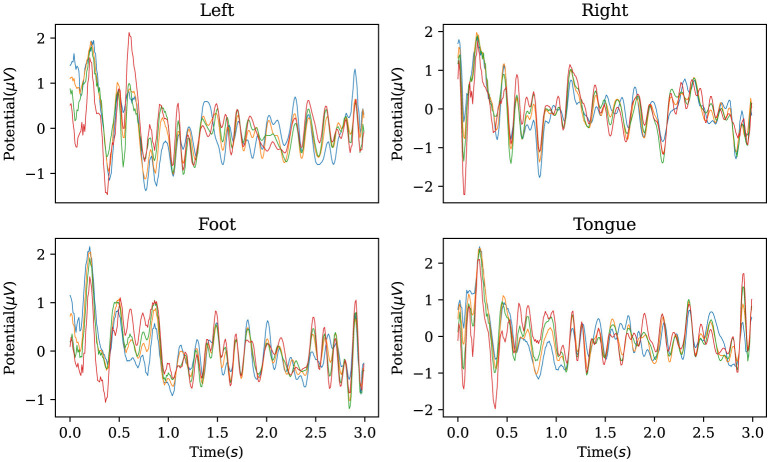
Characteristic waveforms of four types of motor imagery tasks restored using the inverse AMM and transposed convolutional decoder on BCI Competition IV IIa. To be interpretable, the waveforms generated by the model should be similar to the real event-related potentials.

**Figure 6:**
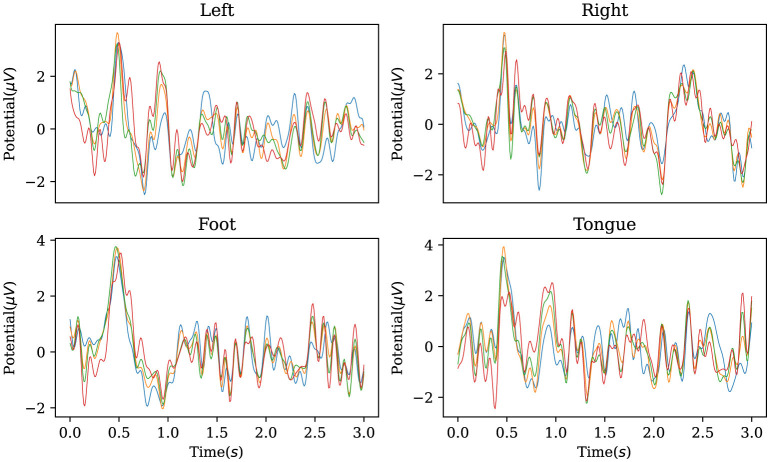
Event-related potentials of four types of motor imagery tasks on BCI Competition IV IIa.

In addition, the established decoder is used to reconstruct the original EEG data corresponding to the labels in the BCI Competition IV IIa dataset, obtaining characteristic waveforms for the four motor imagery categories. As shown in Figure 5, the reconstructed EEG signals reveal distinct waveforms for each of the four categories. When comparing these waveforms with the ERP from the dataset, as illustrated in Figure 6, one can observe a similarity between the reconstructed waveforms and the ERP. The greater the similarity between these two waveforms, the higher the confidence in the model's precise classification. The R-square index is calculated by quantifying the normalized sum of squared differences between the characteristic waveforms and the ERP. Given the four MI tasks on the BCI Competition IV IIa dataset, the R-square satisfies


(19) $$\begin{array}{l} R^{2}=1-\frac{\sum_{i}{\left({ŷ}_{i}-y_{i}\right)}^{2}}{\sum_{i}{\left(ȳ-y_{i}\right)}^{2}}=0.808, \end{array}$$

where ŷ_*i*_ is the reconstructed waveform, *y*_*i*_ is the ERP, and ȳ is the average. The value of *R*^2^ approaches optimum 1, indicating that the reconstructed waveforms of the model can reflect the ERP of the four motor imagery tasks to a high degree.

### 4.4 Discussion

The AM-MTEEG model shows effectiveness in the EEG classification task under the cross-subject variability, as demonstrated through the above experiments on BCI Competition datasets. By incorporating the bidirectional associative memory network, the AM-MTEEG model enables rapid adaptation onto new subjects once the encoder is pre-trained. For practical BCI systems, the proposed model could fit into new subjects using only a small number of samples, facilitating the application of BCI devices for wide users.

With the development of neuromorphic computing, specialized chips have been developed for deploying spiking neural networks (Davies et al., 2018; Pei et al., 2019). Compared to CPUs and GPUs via the Von Neumann architecture, neuromorphic circuits can be implemented using in-memory computing digital or analog circuits. Such neuromorphic circuits offer advantages such as high parallel efficiency, low energy consumption, and compact size. As indicated in Figure 1, both the two stages of AM-MTEEG entail spiking neural networks. This impulsive neuronal attribute make AM-MTEEG potential candidate for edge-computing scenarios in the healthcare field. Hence, it is a doable approach to developing the domestic and miniaturized uses of BCI devices by embedding the proposed model onto neuromorphic circuits.

## 5 Conclusion

This article has developed AM-MTEEG, a multi-task EEG classification model based on deep learning and impulsive associative memory. The model integrates impulsive neural representations from deep learning with bidirectional associative memory networks to alleviate challenges in BCI, such as high variability and limited data in EEG, and the lack of interpretability in end-to-end deep learning. By resorting to the multi-task learning, AM-MTEEG proceeds each subject's classification task as an independent task and leverages cross-subject training to extract shared features and facilitate feature sharing across subjects. Experimental results show the AM-MTEEG model surpasses state-of-the-art methods on the BCI Competition IV IIa dataset, while minimizing classification performance variance across different samples. Altogether, the AM-MTEEG model holds effectiveness in extracting common EEG features, capturing data variability, and handling cross-subject classification tasks. Future work will focus on integrating associative memory networks with deep convolutional networks as to treat single-subject multi-task or multi-subject multi-task BCI scenarios, as well as exploring the joint learning of Hebbian rules and gradient descent for few-shot EEG decoding.

## Data Availability

Publicly available datasets were analyzed in this study. This data can be found here: https://www.bbci.de/competition/.
